# Health and support service needs of individuals with disability from culturally and linguistically diverse backgrounds: a scoping review protocol

**DOI:** 10.1186/s13643-021-01587-8

**Published:** 2021-01-21

**Authors:** Jacqueline Nhu Quynh Pho, Aidan Christopher Tan, Katrina Chaudhary, Sonia Hines, Caroline Ellison, Vivian Isaac, David Lim

**Affiliations:** 1 School of Health Sciences, Western Sydney University, Campbelltown, Australia; 2 South Western Sydney Clinical School, University of New South Wales, Sydney, New South Wales Australia; 3 Centre for Remote Health: a Joanna Briggs Institute Affiliated Group, Flinders University, Alice Springs, Australia; 4 Justice and Society, University of South Australia, Adelaide, Australia; 5 Flinders University Rural Health SA, Flinders University, Renmark, Australia; 6 Penrith, Australia; 7 Translational Health Research Institute, Western Sydney University, Campbelltown, Australia

**Keywords:** Disability, Culturally and linguistically diverse, Health service, Support service, Meaningful engagement in occupation, Meaningful occupations, Scoping review

## Abstract

**Background:**

All individuals should have the right to engage meaningfully in occupations that meet their aspirations and life goals as well as promote their health and well-being. For individuals with disability, meaningful engagement in occupations is supported by timely, effective, and adaptive health and support services. However, research has revealed multiple barriers preventing utilization of these services by individuals with disability from culturally and linguistically diverse (CALD) backgrounds. This review aims to identify gaps and solutions in health and support services of individuals with disability from CALD backgrounds to meaningfully engage in occupations.

**Methods:**

A scoping review will be conducted in accordance with the Joanna Briggs Institute (JBI) methodology for scoping reviews. A detailed search strategy will be used to search CINAHL, PubMed, Embase, Scopus, PsycInfo, JBI, and Cochrane Library, as well as grey literature in Trove, Mednar, and OpenGrey from January 1974 onwards. Two reviewers will independently screen all citations and full-text articles for eligibility against specific inclusion and exclusion criteria. Potential conflicts will be resolved through discussion. Data will be extracted and presented in a diagrammatic or tabular form accompanied by a narrative summary.

**Discussion:**

The scoping review will present the health and support service needs of individuals with disability from CALD backgrounds and will extend the current reviews as it focuses the engagement in meaningful occupation. Findings from this review have the potential to inform local policy discussions and practice-based disability care.

**Systematic review registration:**

Open Science Framework (10.17605/OSF.IO/HW2FB).

**Supplementary Information:**

The online version contains supplementary material available at 10.1186/s13643-021-01587-8.

## Background

All individuals should have the right to engage meaningfully in occupations that meet their aspirations and life goals as well as promote their health and well-being [1]. Meaningful engagement in occupation or meaningful occupation refers to the range of activities, occupations, or pastimes that individuals engage in that are personally or culturally important to, or valued by the individual and provides enjoyment, a sense of self-worth or identity, belonging, or fulfillment [2, 3]. Research has consistently demonstrated that engagement in meaningful occupations positively impact on health and well-being [2, 4–9]. For example, a systematic review on the well-being of elderly individuals showed well-being to be dependent on and enhanced by a range of occupations that provide meaning and value to their life [10]. Similarly, Eakman et al. depicted meaningful engagement in occupation to be associated with better psychological well-being and health-related quality of life [11], and a critical review provided moderate to strong evidence that occupation has an important influence on health and well-being [12]. A lack of meaningful occupation has negative effects on health and well-being.

However, there are multiple barriers preventing meaningful engagement in occupations for individuals with disability. This includes policy and practices failing to meet the health and well-being needs of people with disability [9, 13], such as the failure to provide for the inherent ongoing support for individuals in one or more major life activity [14, 15]. Specifically, Law et al. reported that for children with physical disability, restricted physical, social, and institutional environments limit a child’s engagement in their meaningful occupations [16].

In Australia, it was reported that in 2018, 4.4 million Australians are living with a disability, of which approximately 23% are from a culturally and linguistically diverse (CALD) background [17]. The National Disability Insurance Scheme (NDIS) is a new scheme implemented in Australia in 2013 with the aim of increasing funding options and access to support through services to individuals living with permanent and significant disability under the age of 65 [18]. An objective of this publicly funded insurance scheme is to facilitate the “development of a nationally consistent approach to the access, and the planning and funding of, supports for people with disability” [19], giving individuals more choice and control over their care. To reduce some of the barriers preventing meaningful engagement in occupations by individuals with disability in Australia, the NDIS provides individualized support packages that allow participants to choose services and supports that are reasonable and necessary to support their life and pursuit of their goals and/or meaningful occupation [18]. However, to be eligible for these packages, the NDIS places the onus of proof on individuals with disability to demonstrate their eligibility [19], relying on the individuals’ underlying health literacy and fluency in English for self-activation and decision-making. This indirect discrimination against individuals with disability from non-English speaking CALD backgrounds may make them less likely to seek out support services and less successful in effectively advocating for their needs [20]. Despite the CALD communities having the same prevalence of disability compared to the mainstream Australian population [21], the utilization of support services by individuals with disability from CALD backgrounds is lower [8, 22]. Specifically, the participation rate of CALD individuals in the NDIS is currently only 7.2%, as opposed to CALD individuals with disability, making up roughly 23% of the disabled population [21, 23]. This is approximately half to one-third the rate of NDIS usage by CALD individuals with disability as compared to participants from non-CALD backgrounds [21]. The disparity in support service utilization may be a product of culturally and/or linguistically inappropriate services, failure to accommodate traditional health practices and beliefs, direct discrimination based on race, or indirect discrimination through unconscious biases [21, 24, 25].

The United Nations *Convention on the Rights of Persons with Disabilities*, ratified by 181 countries, affirms the rights of individuals with a disability to receive equal opportunity and participation in society and the highest standard of health care without discrimination [26]. However, it does not address the direct and indirect cultural and racial discrimination towards CALD groups with disability. In Australia, there are specific legislations such as the *Disability Discrimination Act 1992* and *Racial Discrimination Act 1975*, which make it unlawful to discriminate on the basis of disability and race. Yet in reality, research has revealed that some individuals with disability from CALD backgrounds continue to experience actual and de facto discrimination in accessing and utilizing health and support services [21].

Meaningful engagement in occupations for individuals with disability from CALD backgrounds is largely influenced by both health and support services as they can act as enablers providing care and delivery of resources to improve or maintain function, manage chronic complex conditions, disease, or injury [27, 28]. It is important that the health and support service needs of individuals with disability from CALD backgrounds are met to facilitate their meaningful engagement in occupations. Individuals with disability are reported to require more health and support services than those without disabilities [29]; therefore, it is reasonable to anticipate an increased rate of health and support service utilization for this population. However, research has consistently revealed that CALD groups with disability underutilize these health and support services and that such underutilization is dynamic throughout generations [21, 30]. For example, in regard to health services, research on refugee children and adolescents in South Australia revealed that only 21% of those who reported clinically relevant depression symptoms had accessed mental health services [31]. Of those participants, 90% reported they would not use mental health services due to barriers in lack of culturally appropriate health care. The Federation of Ethnic Communities’ Councils of Australia’s review on older individuals from CALD backgrounds affirmed that the underutilization of health and support services by older individuals from CALD backgrounds was not due to lesser needs but is attributed to barriers such as challenges in understanding the Australian systems of care, ability to successfully navigate the healthcare systems, and lack of access to culturally safe and appropriate services [30].

The Australian findings are not unique. Similarly in the USA, a study exploring the perspectives of Asian immigrant parents with children who have special health care needs reported parents experiencing cultural and language barriers in their understanding and navigation of the health-care system [32]. Mirza et al. concluded that existing service systems in the Midwest failed to meet the disability-related needs of refugees, with participants reporting a lack of knowledge related to disability rights and resources and some health care professionals not considering participants’ cultural traditions [33]. Conversely in New Zealand, Mortensen et al. found that the use of cultural caseworkers for children with disability led to benefits for the children’s family including improved access, increase knowledge about health and support services, and improved relationships with their health services [34].

Due to the heterogeneity of the CALD community and the various types of disability, there does not exist a one size fits all solution. Most of the prominent literature focuses on the multicultural issues encountered by recently arrived refugees and asylum seekers, with other CALD groups being underrepresented [35]. As countries become more culturally and linguistically diverse, it is imperative that health and support systems are responsive to the health and support service needs of individuals with disability from CALD backgrounds so as to facilitate meaningful engagement in occupations.

The aim of this scoping review is to identify and describe the health and support service needs of individuals with disability from CALD backgrounds to aid meaningfully engagement in occupations. A scoping review methodology is chosen for its ability to provide a broad overview of the available evidence and include findings from a broader range of CALD individuals with disability [36, 37].

With the completion of the preliminary search on CINAHL in January 2020, no studies were found which discussed the influence of health and support services on meaningful engagement in occupation or what makes occupations meaningful, specific to individuals with disability from CALD backgrounds. Studies referring to other population groups such as individuals with dementia living in residential aged care [38], and engagement in community health programs by non-specific disadvantaged populations [39] were found.

### Review questions

The specific review questions that will be guiding this study are as follows:

1. What are the health and support service needs of individuals with disability from CALD backgrounds to meaningfully engage in occupation?
2. What are the gaps in existing health and support services of individuals with disability from CALD backgrounds?

## Methods

### Study design

The proposed scoping review will be conducted in accordance with the JBI methodology for scoping reviews [40], to assess and synthesize the evidence in published and unpublished literature on individuals with disability from CALD backgrounds. The present scoping review protocol has been registered with the Open Science Framework (registration number: osf.io/hw2fb) and is being reported in accordance with the reporting guidance provided in the Preferred Reporting Items for Systematic Reviews and Meta-Analyses for Protocols (PRISMA-P) statement [41] (see checklist in Additional file 1). The proposed review will be reported in accordance with the reporting guidance provided in the Preferred Reporting Items for Systematic Reviews and Meta-analyses extension for Scoping Reviews (PRISMA-ScR) [42]. Any amendments made to this protocol when conducting the study will be outlined and reported in the final manuscript.

### Eligibility criteria

#### Participants

This scoping review will consider all published and unpublished studies relevant to individuals with disability from CALD backgrounds and their health and support service needs. Individuals from CALD backgrounds are defined mainly by their country of birth, language spoken at home, or characteristics including year of arrival in the adopted country and parents’ country of birth [43].

Disability is defined as the interaction between an individual’s impairment in their body structure or function and their personal and environmental factors that lead to activity limitations and participation restrictions, and prevent meaningful engagement in occupations [44].

#### Concept

The review will consider all studies that describe the health service and health service needs related to individuals with disability from CALD backgrounds. These services are practices that assess, document, maintain, or improve an individual’s health, treat and diagnose illness or disability, or prescribe medication [45].

Studies that describe support service and support needs related to disability will be considered. These services include government services that provide income support to individuals with disability and provision of services and/or funds to organizations to carry out services [46].

Studies that describe meaningful engagement in occupations at a healthcare level will be considered. Meaningful engagement in occupations is the degree to which an individual finds their occupations to be worthwhile, important, and in line with their values and sense of self [3, 11]. Occupation refers to a wide range of activities that individuals “need to, want to, are expected to do” [3] that are worthwhile, important, and compatible with their values and sense of self, ultimately bringing meaning to their life [2, 3, 12].

#### Context

Research conducted in primary and secondary health and support care setting will be considered. Primary care is usually the first contact an individual has with the health system and covers the majority of an individual’s health needs, and delivers community-based care by various health professionals [37, 47, 48]. Secondary care requires more specific knowledge, skills, and equipment and is provided by a specialist or hospital upon referral by a primary care professional [49].

### Study types

This scoping review will consider experimental and quasi-experimental studies, analytical observational studies, descriptive observational studies, qualitative studies, systematic reviews, and text and opinion papers that meet the inclusion criteria. Studies published in the English language will be included. Studies published since January 1974 will be included in order to be comprehensive and attempt to “cover the field”. The index year was chosen as it was the year after the *Immigration Restriction Act 1901* was definitively abolished. This Act restricted immigration of people of non-European ethnic origin to Australia; the abolition of this legislation removed the direct discrimination of individuals based on race.

### Search strategy

A three-step search strategy will be undertaken [40]. An initial search strategy was devised in consultation with a librarian (KC) and employed on CINAHL (EBSCOhost) in January 2020 to identify relevant articles, the text words contained in the titles and abstracts of relevant articles, and the index terms used to describe the articles to develop a full search strategy (see Additional file 2). Initial keywords include disability, culturally and linguistically diverse, multicultural, culturally diverse, linguistically diverse, ethnic minority, minority group, immigrant, migrant, health service, support service, and disability service. A second search will be undertaken including all identified keywords and index terms, which will be adapted for each database. The databases to be searched include PubMed, Embase (Ovid), Scopus, PsycInfo (EBSCOhost), and Johanna Briggs Institute and Cochrane Library, as well as searching for grey literature in Trove, Mednar, OpenGrey, and Google Scholar. Boolean operators and wildcards were applied to search terms to ensure a comprehensive search. Thirdly, the reference list of identified reports and articles will be searched for additional sources. The final search strategies will be reported in the final scoping review report.

### Study selection

This review will include studies relevant to individuals with disability from CALD backgrounds and their health and support service needs. Following the search, all identified citations will be collated and uploaded into Endnote X9 (Clarivate Analytics, PA, USA), and duplicates will be removed. Prior to each stage of screening, reviewers will pilot the eligibility criteria on a random sample of 20 titles/abstracts and 5 full-text studies, with further pilot rounds conducted on an as-needed basis. Titles and abstracts will be screened by at least two independent reviewers (JP, DL, AT, KC) for assessment against the inclusion criteria for the review. An initial calibration will be conducted on 5 randomly selected articles to ensure high inter-rater agreement. The full text of selected literature will be retrieved and assessed in detail against the inclusion criteria (see Additional file 3). Full-text papers that do not meet the inclusion criteria will be excluded, and reasons for exclusion will be provided in an appendix in the final scoping review report. The results of the search will be reported in full in the final report and presented in a PRISMA flow diagram [42] (see Additional file 4). Any disagreement that arises between the reviewers will be resolved through discussion and consensus.

### Data extraction

Data will be extracted from papers included in the scoping review as free-text variables using a modified JBI data extraction instrument [40] (see Additional file 5) by at least two reviewers (JP, DL, AT). The data extraction instrument was pilot tested on five eligible studies retrieved from the initial pilot-literature search conducted in January 2020. Further piloting will be conducted on an as-needed basis. Microsoft Excel will be used to manage the data extracted including the citation and study information (author, year of publication, aim, study population, study design, setting, and methodology), as well as results relevant to the scoping review (type of CALD groups, type of disability, micro-meso-macro system factors, and relevant key findings on the health and support services needs and gaps in existing services). Where required, authors of papers will be contacted to request missing or additional data. Throughout the extraction process, reviewers will meet and compare findings to ensure reliability and reproducibility among data collection approaches. Disagreement or discrepancies between reviewers, during any phase of the review process, will be recorded, and an independent reviewer (SH and VI) will be an arbiter, if necessary.

### Quality appraisal

Quality appraisal will be undertaken independently by two reviewers for all included studies using the appropriate JBI appraisal tools [50] to assess the methodological quality of the studies and ROBINS-I tool for assessing the risk of bias in observation studies [51]. Risk of bias will be assessed independently, in duplicate, by two reviewers. Any discrepancy, including the quality appraisal of observational studies using the JBI and ROBINS-I tools, will be discussed between reviewers to obtain consensus. A kappa coefficient will be obtained. Given the aim of the scoping review to capture the breadth of available literature, the function of the quality appraisal was not selective but rather descriptive and aid in data analysis and interpretation, especially in the context of gaps in the evidence base [52]. Thus, all studies will remain included. Random audit of five included studies will be conducted by SH and VI.

### Data presentation and analysis of results

To illustrate and summarize the main findings, the results of the scoping review will be presented, where appropriate, in a tabular form or as a diagram in a manner that aligns with the objective of this scoping review. A narrative summary will accompany the tabulated results and will describe how the results relate to the review objective and questions [42]. The results will be classified under key conceptual categories that will be obtained during the data extraction process. Common key health and support service needs of individuals with disability from CALD backgrounds to meaningfully engage in occupation identified through the review will be presented as a summary to help illustrate the unique challenges and gaps in existing health and support services, and provide information on how policy may be improved for better engagement in meaningful occupation.

A deductive content analysis framework will be employed to summarize and search for the gaps in health and support services and needs of individuals with disability from CALD backgrounds in accordance with the JBI scoping review methodology [40]. The framework will be used to organize findings from the studies within the individual (micro), interpersonal (meso), organizational (exo), community (macro), and public policy (chrono) level system. Frequency (proportion) of barriers and enablers, patients’ quality of life (based on patients’ score in scale), and satisfaction (based on patients’ score in scale) from the quantitative analysis may be tabulated. Qualitative research findings will, where possible, be pooled using JBI-QARI. This will involve the aggregation or synthesis of findings to generate a set of statements that represent that aggregation, through assembling the findings rated according to their quality and categorizing these findings on the basis of similarity in meaning.

## Discussion

Since our preliminary literature search identified a paucity of publications pertaining to individuals with disability from CALD backgrounds, a scoping review was chosen for its utility in mapping major concepts across a diversity of literature to provide a descriptive overview of the degree, scope, and nature of research activities in a broad topic area and identify gaps in evidence [40, 52]. It is expected that the findings of this review will provide evidence of the health and support service needs of individuals with disability from CALD backgrounds. Furthermore, gaps in existing health and support services of individuals with disability from CALD backgrounds will be described. The review will extend and progress the current knowledge on caring for individuals with disability as it focuses on CALD communities and engagement in meaningful occupation. This protocol is co-designed with CALD stakeholders (VI, DL, KC and JP), individual with disability (CE), and caring responsibility (DL); the Authors have worked in this field. Results of the review will be disseminated through a peer-reviewed publication published in the public domain, will inform subsequent studies in this program of research and has the potential to inform local policy discussions and practice-based disability care. Furthermore, through publishing this research protocol, we encourage practitioners, scholars, researchers, policymakers, and consumers to start the conversation and community of practice on caring for and working with individuals with disability from diverse cultural and linguistic backgrounds, so that health and support services are inclusive and responsive to needs.

This scoping review is constrained to Australian studies and papers published in English, therefore, limits the validity of the findings. Nevertheless, to our knowledge, this scoping review is the first time the health and support service needs of individuals from CALD backgrounds living with disability and the gaps in the current system have been reviewed since the introduction of the publicly funded NDIS. It is the intention that this scoping review will inform future program of research such as a sequential qualitative stakeholders’ interviews to validate and explain the findings from this scoping review.

## Supplementary Information

**Additional file 1.** PRISMA-P 2015 Checklist.

**Additional file 2.** Search strategy for CINAHL (EBSCOhost).

**Additional file 3.** Inclusion and exclusion criteria.

**Additional file 4.** PRISMA 2009 Flow Diagram.

**Additional file 5.** Data instruction instrument.

## Data Availability

All data and materials generated or analyzed are included in this article.

## Abbreviations

CALD: Culturally and linguistically diverse
JBI: Joanna Briggs Institute
NDIS: National Disability Insurance Scheme
CRPD: Convention on the Rights of Persons with Disabilities
PRISMA: Preferred Reporting Items for Systematic Reviews and Meta-analyses
PRISMA-P: Preferred Reporting Items for Systematic Review and Meta-Analysis Protocols
PRISMA-ScR: The Preferred Reporting Items for Systematic reviews and Meta-Analyses extension for Scoping Reviews
