# Supplementary material for: Health and support service needs of individuals with disability from culturally and linguistically diverse backgrounds: a scoping review protocol
Source: Syst Rev. 2021 Jan 21;10:34. doi: 10.1186/s13643-021-01587-8 (PMC7819343; doi:10.1186/s13643-021-01587-8)
Supplement: Supplementary file 2 — Additional file 2. Search strategy for CINAHL (EBSCOhost). [file 13643_2021_1587_MOESM2_ESM.docx]

Search strategy for CINAHL (EBSCOhost)

| # | Query | Results |
| --- | --- | --- |
| S1 | "CALD" OR "culturally and linguistically diverse" OR "migrant*" OR (MH "Transients and Migrants") OR (MH "Ethnic Groups") OR (MH "Immigrants") OR (MH "Cultural Diversity") OR "multicultural*" OR (MH "Multilingualism") OR "multilingual*" OR "cultural* divers*" OR "linguistic* divers*" OR "ethnic minorit*" OR (MH "Minority Groups") OR "minority group" OR "immigrant*" | 88,705 |
| S2 | (MH "Disabled+") OR "disabled" OR "disabilit*" | 191,305 |
| S3 | S1 AND S2 | 2,675 |
| S4 | (MH "Health Services") OR (MH "Health Services for Persons with Disabilities") OR (MH "Health Services Needs and Demand") OR "health service*" OR "disability service*" OR "support service*" OR "healthcare service*" OR (MH "Health Care Delivery") OR "health care delivery" OR "health service* utili*" OR "health service* need*" | 346,570 |
| S5 | (MH "Primary Health Care") OR "primary health care" OR "PHC" OR (MH "Secondary Health Care") OR "secondary health care" OR "SHC" OR "primary support care" OR "secondary support care" OR "primary care" OR "secondary care" | 113,631 |
| S6 | S4 OR S5 | 432,633 |
| S7 | S3 AND S6 | 635 |
