# Supplementary material for: Health and support service needs of individuals with disability from culturally and linguistically diverse backgrounds: a scoping review protocol
Source: Syst Rev. 2021 Jan 21;10:34. doi: 10.1186/s13643-021-01587-8 (PMC7819343; doi:10.1186/s13643-021-01587-8)
Supplement: Supplementary file 3 — Additional file 3. Inclusion and exclusion criteria. [file 13643_2021_1587_MOESM3_ESM.docx]

| Inclusion | Exclusion |
| --- | --- |
| Individuals from CALD backgrounds (37). | Studies not in English. |
| Individuals with disability (38). |  |
| Health service or support service (39). |  |
| Meaningful engagement in occupation/meaningful occupation (1). |  |
| Conducted in Australia or looked at Australian studies. |  |
| 1974 to March 2020. |  |

Inclusion and exclusion criteria
