# Supplementary material for: Health and support service needs of individuals with disability from culturally and linguistically diverse backgrounds: a scoping review protocol
Source: Syst Rev. 2021 Jan 21;10:34. doi: 10.1186/s13643-021-01587-8 (PMC7819343; doi:10.1186/s13643-021-01587-8)
Supplement: Supplementary file 5 — Additional file 5. Data instruction instrument. [file 13643_2021_1587_MOESM5_ESM.docx]

Data extraction instrument

| **Study Details and Characteristics** |  |
| --- | --- |
| Study design |  |
| Study citation details (e.g. author/s, date) |  |
| Country of CALD community |  |
| Context/setting |  |
| Type of CALD groups |  |
| Type of disability |  |
| Participants and demographics (details e.g age/sex and number) |  |
| Study population and sample size (if applicable) |  |
| Aims/purpose |  |
| Methodology |  |
| Methods |  |
| Comments on quality appraisal |  |
| Intervention type, comparator and details of these (e.g. duration of the intervention) (if applicable) |  |
| **Details/Results extracted from study** (in relation to the concept of the scoping review): (i) health and support needs, and (ii) gaps in existing services | |
| Outcomes and details of these (e.g. how measures) (if applicable) |  |
| Key findings/themes that relate to the scoping review question/s. |  |
| Micro-meso-macro system factors |  |
